# Comparing ataxias with oculomotor apraxia: a multimodal study of AOA1, AOA2 and AT focusing on video-oculography and alpha-fetoprotein

**DOI:** 10.1038/s41598-017-15127-9

**Published:** 2017-11-10

**Authors:** L. L. Mariani, S. Rivaud-Péchoux, P. Charles, C. Ewenczyk, A. Meneret, B. B. Monga, M.-C. Fleury, E. Hainque, T. Maisonobe, B. Degos, A. Echaniz-Laguna, M. Renaud, T. Wirth, D. Grabli, A. Brice, M. Vidailhet, D. Stoppa-Lyonnet, C. Dubois-d’Enghien, I. Le Ber, M. Koenig, E. Roze, C. Tranchant, A. Durr, B. Gaymard, M. Anheim

**Affiliations:** 1 APHP, Pitié-Salpêtrière University Hospital, Department of Genetics, F-75013, Paris, France; 2 APHP, Pitié-Salpêtrière University Hospital, Department of Neurology, F-75013, Paris, France; 3 Sorbonne Universités, Université Pierre et Marie Curie Paris 06, Inserm U1127, CNRS UMR 7225, UM 75, ICM, F-75013, Paris, France; 4 Université de Lubumbashi, Faculté de Médecine et Ecole de Santé Publique, Département de Santé Publique, Lubumbashi, Democratic Republic of the Congo; 5 Département de Neurologie, Hôpital de Hautepierre, CHU de Strasbourg, Strasbourg, France; 6 Institut de Génétique et de Biologie Moléculaire et Cellulaire (IGBMC), INSERM-U964/CNRS-UMR7104/Université de Strasbourg, Illkirch, France; 7 Fédération de Médecine Translationnelle de Strasbourg (FMTS), Université de Strasbourg, Strasbourg, France; 8 AP-HP, Département de Neurophysiologie, Département DéPAS, Hôpital Saint-Antoine, Paris, France; 9 Institut du Cerveau et de la Moelle épinière, ICM, F-75013 Paris, France; 10 Inserm U 1127, CNRS UMR 7225, Sorbonne Universités, UPMC Univ Paris 06 UMR_S1127, F-75013 Paris, France; 11 AP-HP, Pitié-Salpêtrière University Hospital, Fédération de Neurophysiologie Clinique, Paris, France; 12 Centre de Référence Neuromusculaire Grand Est (CERNEST), Hôpitaux Universitaires, Strasbourg, France; 13 Department of Tumor Biology, Institut Curie, Paris, France; 14 Institut Curie, INSERM U830 Paris, France; Sorbonne Paris Cité, Université Paris Descartes, Paris, France; 15 Laboratoire de Génétique de Maladies Rares, EA7402, Institut Universitaire de Recherche Clinique, Université de Montpellier, EA 7402, CHU Montpellier, 34093 Montpellier, France; 16 APHP, Pitié-Salpêtrière University Hospital, Department of Clinical Neurophysiology, F-75013 Paris, France

## Abstract

Whether the recessive ataxias, Ataxia with oculomotor apraxia type 1 (AOA1) and 2 (AOA2) and Ataxia telangiectasia (AT), can be distinguished by video-oculography and alpha-fetoprotein level remains unknown. We compared 40 patients with AOA1, AOA2 and AT, consecutively referred between 2008 and 2015 with 17 healthy subjects. Video-oculography revealed constant impairments in patients such as cerebellar signs, altered fixation, impaired pursuit, hypometric saccades and abnormal antisaccades. Horizontal saccade latencies could be highly increased reflecting oculomotor apraxia in one third of patients. Specific distinctive alpha-fetoprotein thresholds were determined for AOA1 (7–15 µg/L), AOA2 (15–65 µg/L) and AT (>65 µg/L). Early age onset, severe walking disability, movement disorders, sensori-motor neuropathy and cerebellar atrophy were all shared. In conclusion, alpha-fetoprotein level seems to permit a distinction while video-oculography does not and therefore is not mandatory, even if an appropriate oculomotor examination remains crucial. Our findings are that AOA1, AOA2 and AT form a particular group characterized by ataxia with complex oculomotor disturbances and elevated AFP for which the final diagnosis is relying on genetic analysis. These findings could guide genetic analysis, assist reverse-phenotyping and provide background for the interpretation of the numerous variants of unknown significance provided by next-generation sequencing.

## Introduction

Autosomal recessive cerebellar ataxias (ARCAs) are clinically and genetically heterogeneous disabling inherited neurodegenerative disorders responsible for cerebellar along with other neurological and systemic signs^1^.

A group of ARCAs due to DNA repair deficiency, RNA termination or maturation deficiencies, or both, has emerged during the past fifteen years. They include ataxia with oculomotor apraxia type 1 (AOA1) due to mutation in *APTX* ^2^, ataxia with oculomotor apraxia type 2 (AOA2) caused by mutations in *SETX* ^3^, ataxia telangiectasia (AT) due to mutation in *ATM* ^4^, and the rarer AT like disorder (ATLD) linked to mutations in *MRE11*.

AOA1 patients have early-onset cerebellar ataxia, hypometric horizontal saccades, sensorimotor neuropathy, optional hypoalbuminemia and common intellectual deficiency^5,6^. Whether AOA1 patients experience genuine oculomotor apraxia (OMA, defined as saccades of elevated latency and optional hypometric saccades^7^) or not remains questionable. AOA2 patients develop cerebellar ataxia in the second decade of life, sensorimotor neuropathy, occasional OMA, strabismus, chorea, dystonia and elevated alpha-fetoprotein (AFP) serum levels^8–12^. Patients with typical AT, the second most frequent recessive ataxia after Friedreich ataxia (FRDA), have progressive cerebellar ataxia in early childhood, typical OMA, oculo-cutaneous telangiectasia, immunodeficiency inducing recurrent infections, increased cancer risk and elevated AFP serum level^13,14^.

AOA1 and AOA2 and AT are considered to share several clinical and molecular characteristrics^1^ even if their individual oculomotor features are not clearly elucidated. Apart from a few studies including oculographic recordings of a single ataxia^5,10,15,16^, specific oculomotor abnormalities of AOA1, AOA2 and AT have never been systematically compared and whether they may be distinguished through oculomotor findings is presently unknown just as whether video-oculography (VO) and biomarkers such as AFP and albumin serum level can assist diagnosis of AOA1, AOA2 and AT in clinical practice. Our aim was to assess whether AOA1, AOA2 and AT could be distinguished from one another by VO and to identify specific AFP thresholds in order to further evaluate the need for VO and AFP in diagnosis. In a controlled study, we compared the clinical, biochemical, video-oculographic, imaging and neurophysiological features of AOA1, AOA2 and AT.

## Results

### Patient Inclusion

**Figure 1:**
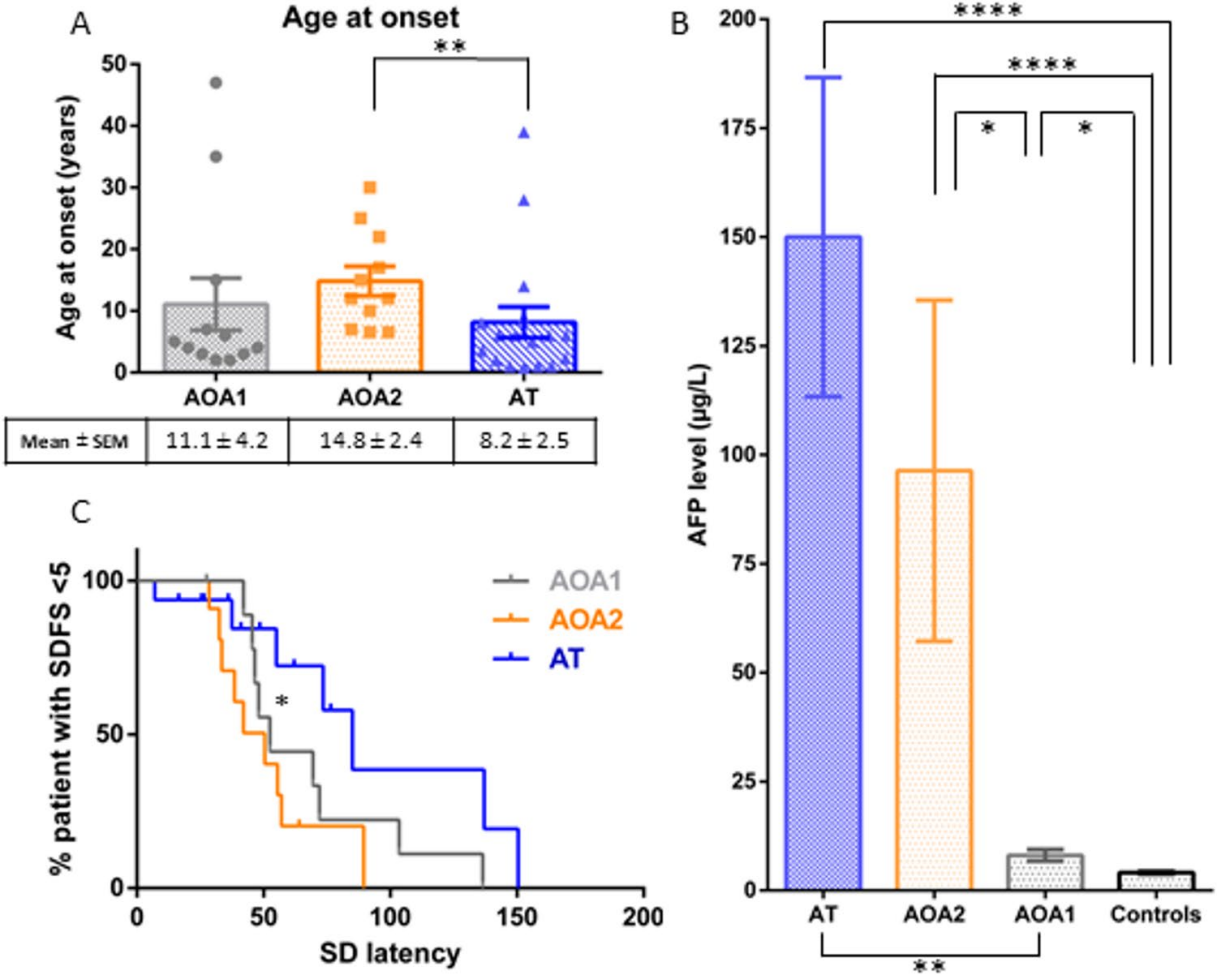
Ages at onset (1A), AFP serum levels (1B) and variability of latencies in respect of disease progression (SDFS) (1C) in AOA1, AOA2 and AT patients. Individual symbols (dots, triangles, squares) represent each patient’s age at onset (1 A), bar graphs represent the mean; upper and lower error bars represent standard error of the mean (1 A and 1B). *p < 0.05; **p < 0.01 and ****p < 0.0001 significant differences. Abbreviations: AFP: Alpha-fetoprotein; AOA1: ataxia with oculomotor apraxia type 1; AOA2: ataxia with oculomotor apraxia type 2; AT: Ataxia Telangiectasia;; SDFS: Spinocerebellar degeneration functional score.

Over a 7-year period (December 2008–April 2015), 40 consecutive cases (22 males, 18 females), with genetically proven AOA1 (n = 12), AOA2 (n = 11) and AT (n = 17), were compared with 17 healthy control subjects (10 females, 7 males). Mutations of each individual and age at time of VO are presented in Supplementary Table 1. Case demographics (Fig. 1A, Supplementary Table 2), clinical characteristics (Table 1), functional disability (Table 1, Supplementary Figure), MRI and Nerve conduction study (Supplementary Table 3), are further described in the Supplementary Results section.

**Table 1:** Clinical characteristics, evolution and functional disability of AOA1, AOA2 and AT patients.

n=	AOA1	AOA2	AT	*p*
	12	11	17	
Age at onset (y)	4.5 [2–47]	12 [6.5–30]**	6 [0.8–39]**	** < 0.01
Age at last follow up (y)	37 [18.1–59.2]	37.8 [22.1–52.3]	32.9 [25.7–56.2]	0.9772
DD at last follow up	23.5 [5–42]	25.3 [9.7–41]	25 [13.8–53.9]	0.634
**Ataxia and functional disability**				
Cerebellar ataxia	12/12 (100)	11/11 (100)	16/16 (100)	
Dysarthria	12/12 (100)	10/10 (100)	16/16 (100)	
Nystagmus	6/10 (60)	6/6 (100)	10/16 (63)	0.2157
SARA at time of VO	23.5 [12–35]	22 [17.5–30]	20.3 [7.5–33]	0.4418
SDFS at time of VO	5 [3–6]	6 [3–6]	4 [1–7]	0.4639
**Associated signs**				
Areflexia	12/12 (100)	11/11 (100)	14/16 (88)	
Vibration sense loss	11/11 (100)	9/9 (100)	7/10 (70)	
Muscle wasting	3/7 (42.9)	6/11 (54.5)	8/16 (50)	1
Motor deficit	6/11 (54.5)	7/11 (63.6)	10/17 (58.8)	1
Pes cavus	3/7 (42.9)	3/11 (27.3)	3/8 (37.5)	0.7915
Scoliosis	1/6 (20)	2/9 (22.2)	4/15 (26.7)	0.8808
Pyramidal signs	2/11 (18.2)	2/11 (18.2)	6/17 (35.3)	0.5855
Telangiectasia	0	0	5/10 (50)	
Hearing loss	0	0	1/7 (14)	
Recurrent infections	0	0	5/17 (29)	
**Movement Disorders**				
Dystonia	4/11 (36.4)*	10/11 (90.9)*	14/17 (82.4)	* < 0.05
Chorea	6/11 (54.6)	5/11 (45.5)	4/17 (23.5)	0.2643
Myoclonus	2/11 (18.2)^a^	1/10 (10) ^a^	11/17 (64.7)	^a^ < 0.05
Tremor	4/11 (36.4)	5/10 (45.5)	2/15 (13.3)	0.2113
Parkinsonism	1/11 (9.1)	0	5/15 (33.3)	0.0557
**Evolution and severity**				
SARA/DD at time of VO	1 [0.6–2.4]	0.9 [0.6–1.9]	0.9 [0.2–1.8]	0.4604
SDFS/DD at time of VO	0.21 [0.1–0.6]	0.24 [0.1–0.4]	0.20 [0.02–0.4]	0.2888
Median age to wheelchair (y)	33.1	40	44	0.9021
Median DD to wheelchair (y)	23	20	42	0.6115
% in wheelchair at age 15	8.3	0	29.4	0.077

Categorical variables are expressed as the ratio of the number of patients presenting the symptom to the total number of patients assessed and as percentages [n/N (%)]; continuous variables as median [range]. Median age and disease duration to wheelchair were calculated by the Kaplan–Meier method.

The SARA and SDFS rates of decline (points per year; SARA/DD and SDFS/DD), defined as respectively SARA and SDFS scores (maximum - minimum)/disease duration, are indexes of disease progression.

*Significant differences after Bonferroni-Holm post-test between the AOA1 and AOA2 groups.

**Significant differences after Bonferroni-Holm post-test between the AOA2 and AT groups.

^a^Significant differences after Bonferroni-Holm post-test relative to the AT group.

Abbreviations: AOA1: ataxia with oculomotor apraxia type 1, AOA2: ataxia with oculomotor apraxia type 2, AT: Ataxia-Telangiectasia; DD: Disease Duration; SARA: Scale for the Assessment and Rating of Ataxia; SDFS: Spinocerebellar degeneration functional score; VO: video-oculography; y: years.

### Biomarkers

AFP serum levels in ataxic patients were significantly increased in the AOA1, AOA2 and AT groups relative to those in 100 control patients without cerebellar ataxia. An elevated AFP over 7 µg/L had a sensitivity (Se) of 82%, a specificity (Sp) of 90%, a positive predictive value (PPV) of 76% and a negative predictive value (NPV) of 93% in distinguishing ataxic patients (AOA1, AOA2 and AT) relative to the controls. AFP levels were significantly higher in AOA2 and AT than in AOA1 patients (Supplementary Table 3; Fig. 1B). Thresholds of AFP levels permitting distinction between ataxic patients were determined. AFP levels of 7 to 15 µg/L had Sp of 93%, PPV of 78% and NPV of 86% for AOA1 relative to AOA2 and AT patients. AFP levels of 15 to 65 µg/L had Sp of 86%, PPV of 64% and NPV of 89% for AOA2 relative to AOA1 and AT patients. AFP levels over 65 µg/L had Sp of 90%, PPV of 83% and NPV of 73% for AT relative to AOA1 and AOA2 patients.

Albumin serum levels were only decreased in AOA1 being significantly lower than in the AOA2 and AT groups (Supplementary Table 3), while hypoalbuminemia had Sp of 95.5% for AOA1 relative to AOA2 or AT patients.

### Video-Oculography

VO was performed for all 40 ataxic patients and the 17 healthy control subjects. The median ages proved not significantly different. Results are summarized in Table 2 and Fig. 1C; individual results are detailed in Supplementary Table 1.

**Table 2:** Video-oculographic findings in our AOA1, AOA2, AT patients and control group.

n=	AOA1	AOA2	AT	Controls	p
	12	11	17	17	
**Age at VO**	37 [15.4–58.7]	37.6 [21.6–51])	28.6 [22–54.8]	33 [25–38]	0.7744
**SARA**	23.5 [12–35]	22 [17.5–30]	20.3 [7.5–33]	NR	0.4418
**SARA/DD**	1 [0.6–2.4]	0.9 [0.6–1.9]	0.9 [0.2–1.8]	NR	0.4514
**SDFS**	5 [3–6]	6 [3–6]	4 [1–7]	NR	0.4639
**SDFS/DD**	0.21 [0.1–0.6]	0.24 [0.1–0.4]	0.20 [0.02–0.4]	NR	0.3023
**Fixation**					
**SWJ**	4 (33.3)	7 (63.6)**	5 (31.3)	0	** < 0.01
**DBN**	3 (25)	7 (63.6)**	6 (42.9)*	0	* < 0.05 ** < 0.01
**GEN**	7 (58.3)**	9 (81.8)****	8 (50)**	0	** < 0.01 **** < 0.0001
**Abnormal fixation**	8 (58.3)^a^ **	11 (100) ^a^ ****	13 (75)****	0	^a^ < 0.05 ** < 0.01 **** < 0.0001
**Saccades**					
**Horizontal**					
- **Latency**					
Mean latency	202.3 [139.5–492]	191.5 [136–354]	198 [160.5–449]	173.5 [138.5–245]	0.1773
SD of latency	50.25 [27.5–136.5]	42.0 [28.5–89.5]	48.5 [16.5–150.5]	33.0 [14.5–103]	0.0643
	All ataxia (AOA1, AOA2, AT): 48.3 [16.5–150.5]^b^			33.0 [14.5–103]	^b^ < 0.05
Increased latency	4 (40)	3 (27.3)	5 (33.3)	3 (18.8)	0.4097
**-Gain/Amplitude**					
**Centrifugal saccades**					
Hypometric	9 (81.8)****	8 (72.7)****	14 (82.4)****	0	**** < 0.0001
Hypermetric	2 (18.2)****	1 (9.1)****	2 (11.8)****	0	**** < 0.0001
**Centripetal saccades**					
Hypometric	5 (45.5)****	3 (27.3)****	8 (47.1)****	0	**** < 0.0001
Hypermetric	4 (36.4)****	8 (72.7)****	6 (35.3)****	0	**** < 0.0001
**-Horizontal velocity**					
decreased	4 (33.3)	2 (18.2)	1 (6.3)	0	< 0.05
**Cerebellar symptoms**	8 (72.7)***	11 (100)****	13 (81.3)****	0	*** < 0.001 **** < 0.0001
Vertical					
- **Vertical velocity** decreased	3 (25)	6 (54.6)**	3 (20)	0**	** < 0.01
**-Upward latency**					
Mean latency	241 [154–326]	221 [133–335]	239 [153–302]*	176 [152–221]*	* < 0.05
SD of latency	All ataxia (AOA1, AOA2, AT): 58 [16–194]			41 [23–81]	0.0864
**- Downward Latency**					
Mean latency	193 [156–263]	185 [131–287]	196 [161–309]	180.5 [147–249]	0.3603
SD of latency	All ataxia (AOA1, AOA2, AT): 47 [12–142]			36 [15–125]	0.1347
**Smooth pursuit** saccadic or abolished	10 (91)	11 (100)	12 (75)	ND	0.4443
**Antisaccades**					
**% of errors**	23.5 [0–77.5]	51.5 [6–80.5]***	45.5 [6–86.5]***	10.5 [0–32]***	*** < 0.001
**number of patients > 15% errors**	5 (62.5)	10 (91)**	12 (92.3)**	5 (33.3)**	** < 0.01

Categorical variables are expressed as numbers and percentages (n(%)); continuous variables as median [range].

^a^Significant differences after Bonferroni-Holm post-test between the AOA1 and AOA2 groups.

*, **, *** and **** Significant differences after Bonferroni-Holm post-test relative to the control group.

^b^Significant differences after unpaired two-tailed Mann Whitney t test between the groups “all ataxia” and “controls”.

The SARA and SDFS rates of decline (points per year; SARA/DD and SDFS/DD), defined as respectively SARA and SDFS scores (maximum - minimum)/disease duration, are indexes of disease progression.

Abnormal Fixation is defined as at least one of: SWJ, DBN or GEN.

Cerebellar symptoms are defined as presenting at least one of: DBN or GEN or Hypermetric horizontal saccades.

Abbreviations: AOA1: ataxia with oculomotor apraxia type 1, AOA2: ataxia with oculomotor apraxia type 2, AT: Ataxia Telangiectasia; DBN: down beat nystagmus; DD: Disease Duration; GEN: gaze-evoked nystagmus; ND: Not done; NR: Not Relevant; SARA: Scale for the Assessment and Rating of Ataxia; SD: Standard Deviation; SDFS: Spinocerebellar degeneration functional score, SWJ: Square Waves Jerks, VO: video-oculography.

**Figure 2:**
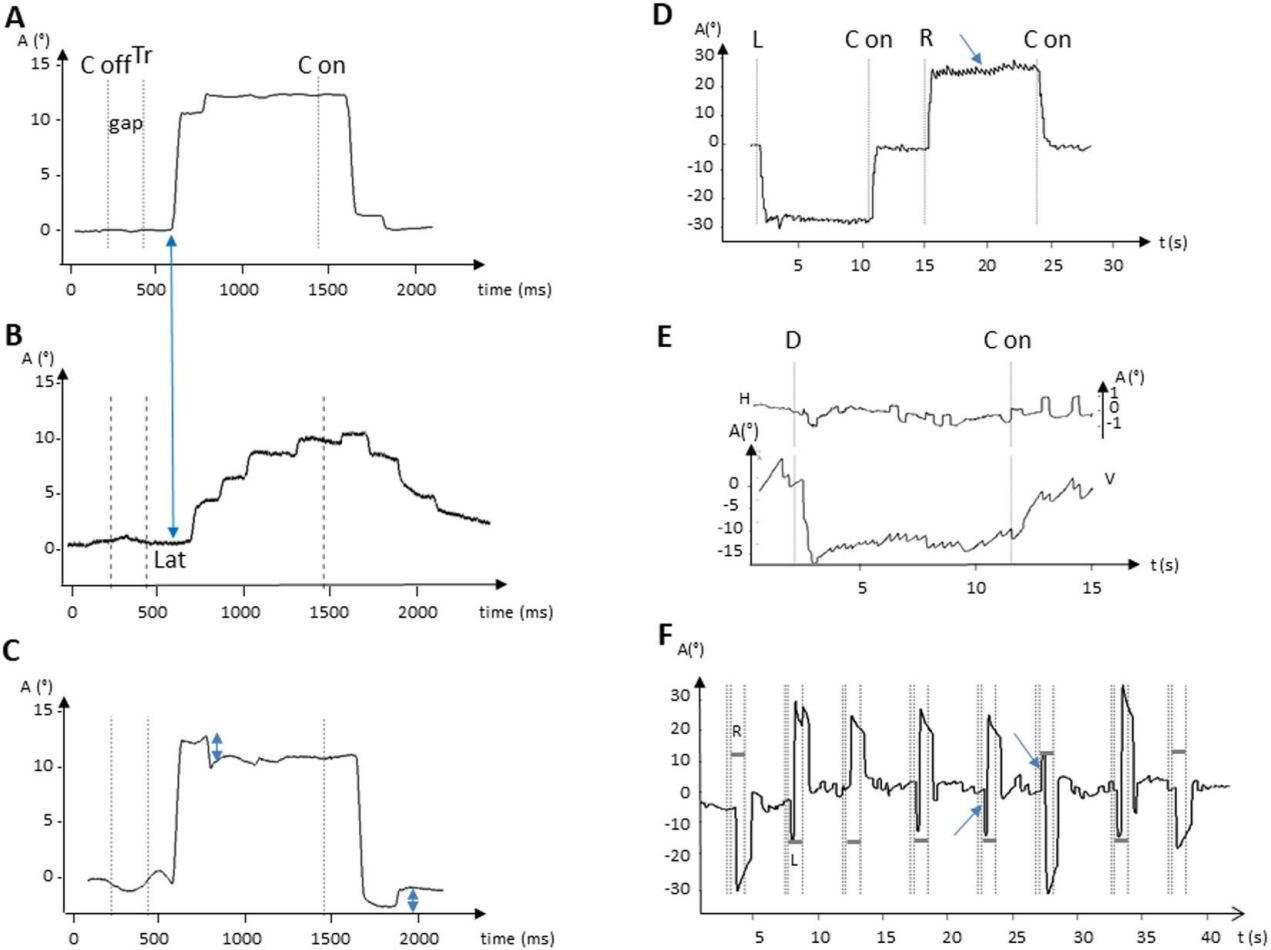
A variety of abnormal eye movements common to AOA1, AOA2 and AT patients found on recordings by Video-Oculography. (**A**) Rightward saccade of a control subject; the blue arrow indicates the beginning of the saccade in the control subject with a normal latency. (**B**) Hypometric rightward saccade with increased latency of an AOA1 patient; the blue arrow indicates the beginning of the saccade in the control with a normal latency. Note how the latency is increased in the patient with a saccade starting later when compared with the control. (**C**) Hypermetric centrifugal and centripetal saccades (double arrows) of an AT patient; (**D**) Gaze-evoked nystagmus (arrow) in an AOA2 patient; (**E**) Square wave jerks (upper panel) and Downbeat nystagmus (bottom panel) of an AOA2 patient. (**F**) Antisaccade task with saccade errors directed to the target (horizontal grey bars = target position; arrows = errors to the left and the right). Abbreviations: A: amplitude; C off: center off; C on: center on; D: Down; H: horizontal trace; L: left; Lat: latency; R: Right; Tr: target on the right; V: vertical trace.

VO revealed complex oculomotor disorders. Various typical abnormal findings in the three groups of patients are illustrated in Fig. 2. Almost all patients displayed cerebellar symptoms with downbeat nystagmus (DBN; Video 1) and/or gaze-evoked nystagmus (GEN; Video 2) or hypermetric horizontal saccades (Video 3) (Table 2). Saccades could often be both hypo- and hypermetric on centrifugal and centripetal saccades. The typical pattern of marked hypometric saccades is shown on Fig. 2B. Fixation is often abnormal, disrupted by either square wave jerks (SWJ), DBN or GEN, occurring more frequently in AOA2 and AT than in AOA1 patients.

In AOA1, AOA2 and AT, horizontal saccade latencies could be greatly increased (defined as > than 229 ms), reaching 492 ms, reflecting OMA though latencies were not increased in all patients, only in 27.3 to 40%.

Variability of saccade parameters was high for each group of ataxic patients as shown by the broad range of mean latencies. This was also true in individual observations during the VO recording sessions for each ataxic patient, as shown by the high SD of latencies (Table 2, Supplementary Table 1). The SD of latencies increases with decreasing age at onset and with higher SDFS (Fig. 1C), this is more significant in AT patients (p < 0.05).

Horizontal and vertical velocities could be decreased in some patients (Table 2). While this pattern occurred more notably in AOA2 patients it was an occasional finding in all three groups. Smooth pursuit was saccadic or abolished in almost all patients. Antisaccades were significantly altered in all three groups, though more frequently in AOA2 and AT relative to the controls.

### Literature Review

**Figure 3:**
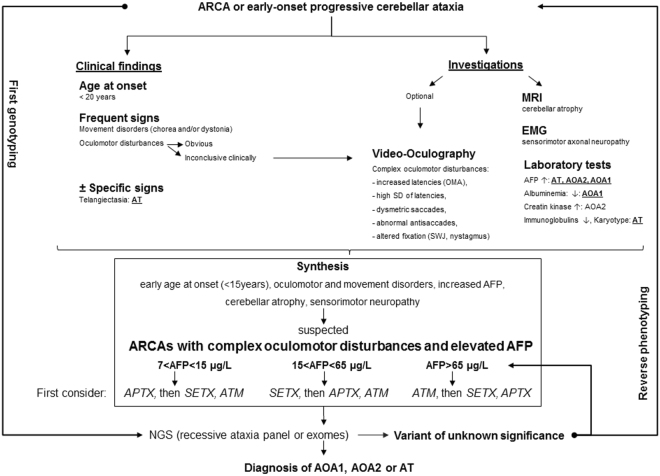
Workup leading to AOA1, AOA2 and AT diagnosis in view of the results of our study and previous reports. Abbreviations: AFP: Alpha-fetoprotein; AOA1: ataxia with oculomotor apraxia type 1, AOA2: ataxia with oculomotor ataxia type 2; ARCA: autosomal recessive cerebellar ataxia; AT: ataxia telangiectasia; EMG: electromyography; NGS: Next-Generation Sequencing; OMA: Oculomotor apraxia; SD: Standrad deviation; SWJ: Square wave jerks.

Supplementary Table 4 presents previous studies reporting oculographic recordings in genetically confirmed AOA1 or AOA2 or AT patients. Only 10 studies reported oculographic recordings in either AOA1 (2 studies with recordings of 3 and 6 patients respectively) or AOA2 (3 studies with 2 to 7 patients recorded) or AT (5 studies with 3 to 33 patients recorded). Some AT patients’ VO results reported here were partly described in Méneret *et al*.^17^. No direct comparison between AOA1, AOA2 and AT was reported in any study. Figure 3 shows the workup leading to AOA1, AOA2 and AT diagnosis in view of the results of our study and previous reports^18^.

## Discussion

We report that AOA1, AOA2 and AT which together should be considered as a particular group among ARCAs, share many features including complex, overlapping oculomotor disturbances, elevated AFP along with sensorimotor axonal neuropathy, chorea and/or dystonia, cerebellar atrophy and severity. Genuine OMA may also be a feature of this group though it is not ubiquitous.

Surprisingly, VO alone is not able to distinguish between AT, AOA1 and AOA2 and is therefore not a tool mandatory to diagnosis in clinical practice, however it can otherwise be helpful in revealing the complex and marked variability in the oculomotor disorders suggestive of this group of three ataxia (Fig. 3).

Constant impairments - in line with previous cases reviewed here (Supplementary Table 4) - are cerebellar signs, altered fixation, impaired pursuit, hypo- and hypermetric saccades and increased antisaccade error rates. Both centrifugal and centripetal saccades should be examined as hypermetric saccades are more often found on centripetal saccades. Altered fixation and dysmetric saccades have functional consequences as daily life activities such as reading^19^ or watching TV will be impaired^20^. Horizontal and vertical velocities can appear decreased in some patients though, in most of them, VO reveals severe hypometria with “multiple step saccades” resulting in a prolonged duration of total gaze shifts which can mimic slow saccades^5^ (Table 2). Despite definite OMA in some patients with greatly increased latencies, the mean latencies of each group are surprisingly not significantly higher than in healthy subjects. This reflects two points: (i) As indicated here and in previous studies, OMA is not automatically present in these ARCAs (Supplementary Table 4); (ii) Findings vary from one patient to another and from one moment to the next, even during the same recording session, as reflected by the higher SD and broad range of latencies. This variability increases with disease severity (Fig. 1C). In clinical practice, the search for saccades of increased latency and a broad range of saccade latency is crucial. VO offers a more accurate means of identifying such features.

Compared with the controls, many oculomotor parameters become altered in AOA1, AOA2 and AT though with no identifiable differences between them. This might be explained by the small sample size, the late stage of disease evolution as shown by their SARA and SDFS scores at recording (ceiling effect), or reflect their very close phenotype. Another limitation may be that examinations were only performed in head-fixed conditions. A head-free condition provides additional interest such as movements that may be associated with horizontal saccades like head thrusts^16^. The various oculomotor perturbations reported here reflect a diffuse cerebellar involvement (including flocculus/paraflocculus, fastigial nuclei, vermis) but also pons, midbrain and parieto-frontal cortices^21^. Antisaccade alterations reflect frontal involvement by increased difficulty in inhibiting automatic responses to the visual targets^21^. Beyond cerebellar failure, AOA1, AOA2 and AT are due to a diffuse central nervous system deterioration.

This is, to our understanding, the first multimodal, controlled study comparing AOA1, AOA2 and AT. Here all patients underwent complete VO recordings whereas previous studies with smaller patient samples, focused specifically on only parts of the oculomotor abnormalities (Supplementary Table 4).

To help in the distinction between AOA1, AOA2 and AT in clinical practice other more subtle features, such as the possibility of AFP distinctive thresholds, may be useful. AFP serum level is increased in all three diseases with good sensibility, specificity, PPV and NPV relative to the controls. As previously reported, almost all AOA2 and AT patients have increased AFP serum levels over the disease course (up to 470 µg/L), higher than AOA1 patients (always below 15 µg/L), which are still above the controls. Therefore, if initially normal, AFP assessments should be repeated during the evolution of an ataxia of unknown etiology. Surprisingly, AOA1 patients often reveal a slightly elevated AFP serum level. This agrees with a previous report of three AOA1 patients^22^. Alternately, other reports of ARCA3/*ANO10* ^23^ and AOA4 due to *PNKP* mutations^24^, have suggested that elevated AFP is not such a specific biomarker. In our study comparative AFP serum levels were able to distinguish between AOA1, AOA2 and AT patients being based on discriminating thresholds with good specificities and predictive values.

Age at onset, considered an important point in ARCAs diagnosis workup^1^, could also be a factor in the differentiation of these ARCAs. Adult forms of AT – corresponding to patients with prolonged survival and/or delayed age at onset^17,25^ – show a significantly lower age at onset than both AOA1 and AOA2. Mean ages at onset reported here are close to previous reports^5,7,9,10^ though we also report a few patients with older ages at onset (Fig. 1A). As further information expands our knowledge of designated phenotypes, particularly in adult forms^17,25,26^, the diagnosis of these disorders must encompass patients with a later onset. This renders the distinction of AT from AOA1 and AOA2 more difficult while further supporting their inclusion into the recessive ataxias with complex oculomotor disturbances and elevated AFP group.

When assessed in adulthood the severity of these three ataxias is comparable and uniform. AT patients are divided into two groups: (i) Patients with an early onset and marked severity who become wheelchair bound after 10 years of disease progression, and (ii) Patients whose disease advances more slowly and which sometimes had a later age at onset, becoming wheelchair bound after decades of evolution (Supplementary Figure)^13,17^. As an adult tertiary center, we assess patients affected by recessive ataxias who survive beyond childhood. The patients displaying such a variant form of AT could be responsible for the peculiar wheelchair-bound survival curve (Supplementary Figure), with a more severe curve for AT during the first stages of the disease; while in the later stages, the curve is less severe for AT than for AOA1 and AOA2.

In conclusion, we have shown that VO is not able by itself to differentiate AOA1, AOA2 and AT and is therefore not mandatory to the diagnosis workup of patients suspected with ARCA, though it is recognized that an appropriate oculomotor examination remains crucial. Increased AFP serum level is a relevant tool for their distinction. AOA1, AOA2 and AT should be considered among ARCAs as a particular group of ataxia with complex oculomotor disturbances and elevated AFP for which the diagnosis relies on genetic analysis. However, clinical and biological investigations are still mandatory before genetic analysis in order to guide the geneticist through the analysis of genetic data, especially under the era of NGS. Our findings broaden the spectrum of ataxia with elevated AFP and will help reverse-phenotyping in cases of first genotyping and in the interpretation of the numerous variants of unknown significance provided by premature NGS (Fig. 3).

## Methods

Patients consecutively diagnosed with genetically proven AT, AOA1 and AOA2 in our tertiary adult movement disorder centers (Pitié-Salpêtrière Hospital, Paris, France and University Hospital of Strasbourg, France), were assessed by comprehensive clinical examination, VO recordings, EMG, imaging and laboratory investigations (Supplementary Methods) between December 2008 and April 2015.

### Video-Oculography

VO was performed in all patients. The subjects were seated in complete darkness facing a screen located 57 cm in front with head and chin supported. Eye movements were recorded with a video-based monocular eye tracker (500 Hz, SMI, Germany). To study the presence of fixation abnormalities such as gaze-evoked nystagmus or downbeat nystagmus, a target was presented in each direction (25° to the left and to the right, 15° up and down), during 8 minutes.

The protocol then continued with a visually guided saccade task (VGST), an antisaccade task (AST) and a smooth pursuit task (SPT). In the VGST, subjects were asked to follow as quickly and as accurately as possible a visual target stepping from a central to a 13° lateral right or corresponding left position. A minimum of 16 trials in each direction was recorded for each subject during each task. A 200 ms blank interval (gap) occurred between central fixation offset and lateral target onset. VGST was also performed with 13° vertical targets. Saccade latency was determined (Eyebrain software). Saccades with latencies below 90 ms or perturbed by blinks were manually discarded. Horizontal saccade latencies with values above the control group mean + 2 SD were considered as increased. Velocity and gain (ratio of initial saccade amplitude on target step), were determined when possible (Eyebrain software) but qualitatively assessed when important background activity (such as nystagmus), resulting in calibration difficulties prevented quantification.

For the AST, the same sequence was used excepting that subjects were instructed not to follow the lateral target but instead to look as quickly as possible in the opposite direction. A minimum of 18 trials in each direction was recorded for each subject during each task. Antisaccade error rate (i.e. the percentage of misdirected antisaccades), was similarly analyzed. In the SPT, subjects were asked to follow as accurately as possible a target that moved horizontally with a slow sinusoidal velocity (two peak velocities performed: 12 and 25°/s). In healthy subjects, very few catch-up saccades occur, while in most severely affected patients, pursuit may be totally replaced by catch-up saccades. In order to provide an evaluation of smooth pursuit, we used an index defined as the percentage of eye displacement performed by catch-up saccades. Smooth pursuit was considered as normal for an index below 25%.

### Control groups for Video-Oculography and AFP serum level

To compare the AOA1, AOA2 and AT patients with the control group, we recorded the ocular movements of the 17 subjects free of any neurological disease.

AFP serum levels were also determined for 100 healthy control subjects and 100 non-ataxic control patients using the immunoanalysis Kryptor Brahms method, as established in the “Laboratoire de Biochimie Générale et Spécialisée” (LBGS) in the University Hospital of Strasbourg, France, as previously described^7^. The measure of AFP level in 100 healthy subjects in applying this method yielded a median value of 3.2 mg/l with a 97.5 percentile at 7 mg/l, which was considered as the upper limit in our study. We also tested for AFP levels one hundred control patients who had no cerebellar ataxia but were affected with one of Parkinson’s disease (n = 37), multiple system atrophy (n = 13), Huntington’s disease (n = 3), dystonia (n = 1), Alzheimer’s disease (n = 11), multiple sclerosis (n = 11), amyotrophic lateral sclerosis (n = 2), peripheral neuropathy (n = 16) and myopathy (n = 6). These patients were not investigated for AOA1, AOA2 and AT mutations.

### Statistical analysis

Patients’ data were analyzed using the statistical software package Statistical Analysis System (SAS) for Windows, release 9.3 (SAS Institute Inc., Cary, NC, USA) and GraphPad Prism software, release 6.0 (Supplementary Methods).

### Standard protocol approvals, registrations, and patient consents

Written informed consent was obtained from the patients (or the parents of minors), before blood sampling and genetic analyses. The study was approved by local ethics committees from the institution APHP, Pitié-Salpêtrière Hospital, Paris, France and University Hospital of Strasbourg, France. All protocols were performed in accordance with the relevant guidelines and regulations.

### Literature Review

The NIH PubMed database was scanned from June 1995 up to June 2016 for reports in English containing oculographic recordings of molecularly confirmed AT, AOA1 or AOA2 patients. Studies reporting exclusively clinical assessment of eye-movement, or with no genetically confirmed AT, AOA1 or AOA2 patients, were discarded.

### Data availability

The datasets generated and/or analyzed during the current study are available in the supplementary material section and from the corresponding author on reasonable request.

## Electronic supplementary material

Supplementary data

Video 1

Video 2

Video 3
