# Supplementary material for: Comparing ataxias with oculomotor apraxia: a multimodal study of AOA1, AOA2 and AT focusing on video-oculography and alpha-fetoprotein
Source: Sci Rep. 2017 Nov 10;7:15284. doi: 10.1038/s41598-017-15127-9 (PMC5681651; doi:10.1038/s41598-017-15127-9)
Supplement: Supplementary file 1 — Supplementary data [file 41598_2017_15127_MOESM1_ESM.doc]

**Supplementary Material to Comparing ataxias with oculomotor apraxia: a multimodal study of AOA1, AOA2 and AT focusing on video-oculography and alpha-fetoprotein**

by L.L. Mariani, S. Rivaud-Péchoux, P. Charles, C. Ewenczyk, A. Meneret, B.B. Monga, M-C. Fleury, E. Hainque, T. Maisonobe, B. Degos, A. Echaniz-Laguna, M. Renaud, T. Wirth, D. Grabli, A. Brice, M. Vidailhet, D. Stoppa-Lyonnet, C. Dubois-d’Enghien, I. Le Ber, M. Koenig, E. Roze, C. Tranchant, A. Durr, B. Gaymard and M. Anheim.

**Supplementary Tables: 4**

**Supplementary Table 1:** Detailed individual video-oculographic recording data, mutation status, SARA and SDFS scores at time of recording of AOA1, AOA2, AT and Control groups**.**

Supplementary Table 1a: Detailed individual mutation status, Age, Disease duration, SARA and SDFS scores at time of recording,

Supplementary Table 1b: Detailed individual video-oculographic recording data

**Supplementary Table 2:** AOA1, AOA2 and AT patient demographics

**Supplementary Table 3:** Investigations: biomarkers, nerve conduction and imaging findings

**Supplementary Table 4:** Literature review of video-oculographic findings in AOA1, AOA2, or AT patients

**Supplementary Figure:** Severity of disease progression in AOA1, AOA2 and AT patients.

**Videos: 3**

**downbeat nystagmus (Video 1) and/or gaze-evoked nystagmus (Video 2) or hypermetric horizontal saccades (Video 3) in ataxic patients**

**Supplementary Results**

**Case Demographics**

Mean age at onset is shown on Figure 1A. supplementary Table 2 describes the demographics of the AT, AOA1 and AOA2 patients.

AT have a significantly lower age at onset than AOA2 patients despite two individual AT with a later age at onset (Figure 1A).

In the three groups, patients’ presenting symptoms were mostly in relation to their cerebellar ataxia: unsteadiness, clumsiness and dysarthria. Initial symptoms such as movement disorders like dystonia could also be the first sign of AT (supplementary Table 2).

**Clinical characteristics and functional disability**

Patients’ clinical characteristics are described in Table 1.

All the patients experienced cerebellar ataxia with a marked functional disability, reflecting the severity of disease progression as shown by the strong correlation between SARA/DD and SDFS/DD ratios in AOA1 (r²=0.87; p <0.0001), AOA2 (r²=0.73; p<0.001) and AT (r²=0.94; p<0.0001) patients. Disease severity (SARA/DD and SDFS/DD ratios) was not significantly different among groups, with a wider range in AT.

Movement disorders including dystonia, chorea, myoclonus, tremor and parkinsonism were often present in all groups. Almost all AOA2 and AT patients showed dystonia, significantly less AOA1 did. AT patients experienced myoclonus more often than AOA1 and AOA2 (Table 1). Up to a third of patients demonstrated parkinsonism (Table 1).

Median age at VO (33.1 to 44 years-old) and disease duration (20 to 42 years) at wheelchair were not significantly different among groups (Table 1, Figure 1B). A third of AT patients became wheelchair bound at age 15.

When searched for, other signs such as neoplasia (n=1), telangiectasia, hearing loss and recurrent infections were only found in AT patients (Table 1).

**Magnetic resonance imaging**

Brain MRI was performed in 83% (n=33/40) of patients with respectively 7/12 AOA1, 11/11 AOA2 and 15/17 AT patients investigated (supplementary Table 3). All MRIs showed obvious cerebellar atrophy.

**Nerve conduction study**

Motor and sensory nerve conduction studies (NCS) were performed in 90 % (n=36/40) of patients. An axonal sensory motor neuropathy was found in a majority (76.5% to 90%) of patients in each group (supplementary Table 3).

**Supplementary Methods**

**Patients**

Patients with genetically proven AOA1, AOA2 and AT who were consecutively referred for VO at two French tertiary adult movement disorders centers (Pitié-Salpêtrière Hospital, Paris and University Hospital of Strasbourg) between December 2008 and April 2015 were included. All patients had a standardized interview and a detailed clinical examination by a movement disorders expert (P.C., E.R., C.E., D.G., C.T., A.D., B.D., A.M., M.V., LL.M. or M.A.).

Age at onset of the disease, disease duration (DD) and age at last examination were noted as well as gender and geographic origin, symptoms at onset, muscle strength, vibratory sense, deep tendon reflexes, plantar reflexes, strabismus, pes cavus, scoliosis, chorea, myoclonus, dystonia, tremor and parkinsonism. Pyramidal tract involvement was retained in case of extensor plantar reflexes and/or both increased and diffused tendon reflexes. We also reviewed the patients’ medical history for infections during childhood or malignancies, and carefully looked for telangiectasia.

Scale for the Assessment and Rating of Ataxia (SARA, from 0 to 40, 40 being the most severe [1]) was used to quantify the severity of ataxia and Spinocerebellar degeneration functional score (SDFS) was used to evaluate the disability stage from 1 to 7 (0: no functional handicap; 1: no functional handicap but signs at examination; 2: mild, able to run, walking unlimited; 3: moderate, unable to run, limited walking without help; 4: severe, walking with one stick; 5: walking with two sticks or a stroller; 6: unable to walk, requiring wheelchair; 7: confined to the bed). SARA and SDFS corrected for DD (SARA/DD ratio and SDFS/DD ratio) were used to evaluate the progression rate of the disease.

For some patients, karyotype was performed.

**Genetic analysis**

*SETX*, *APTX* and *ATM* genes were sequenced as previously described [2–4]. All 24 coding exons of *SETX* (NM_015046.5, Moreira et al., 2004[5]), 7 coding exons of *APTX* (NM_175073.2, Moreira et al., 2001[6]) and 62 coding exons of *ATM* (RefSeq U82828.1) and an average of 30 nucleotides spanning each exon/intron junction were analyzed by direct sequencing of genomic DNA. Sequences were analysed using either the Seqpilot software version 2.0 (JSI medisys, Kippenheim, Germany) or the SeqScape software version 2.5 (Applied Biosystems, Foster City, USA). The search for large gene rearrangements was performed by semi-quantitative PCR, using the Multiplex Ligation dependent Probe Amplification (MLPA) kit (SALSA MLPA KIT P041/P042 ATM or SALSA MLPA kit P316-A1 Recessive ataxias, MRC-Holland, Amsterdam, The Netherlands). In addition, ATM missense mutations were indirectly studied through the response to ionizing radiation (IR) of lymphoblastoid cell lines established from patients carrying a homozygote mutation. For compound heterozygote mutations, the presence of one mutation on each parental allele (in trans) was verified when parental DNA was available. In addition, 200 healthy European individuals and/or ExAC database (<http://exac.broadinstitute.org/>) were used as control for sequencing. As nucleotide variations can lead to splicing abnormalities, the genomic sequence environment of each DNA variant was analysed using Splice Site Prediction by Neural Network (NNSPLICE available at http://www.fruitfly.org/seq_tools/splice.html), MaxEntScan (MES available at <http://genes.mit.edu/burgelab/maxent/Xmaxentscan_scoreseq.html>) (Houdayer et al 2008[7]). All missense mutations with unknown biological effects were considered to have a likely pathogenic effect when at least 2 of the following criteria were present: carrier frequency of less than 1% in a series of controls, location in a domain that is required for function (especially the phosphatidylinositol 3-kinase and FRAP-ATM-TRRAP domains for *ATM*, the SEN1_N and 7-motif helicase domains for *SETX*, and the Histidine-triad [HIT] domain for *APTX*), or a high score from the Align-GVGD algorithm, which quantifies amino acid changes and their conservation among species (Tavtigian et al 2008[8]). The IR cellular response was studied through KAP1 phosphorylation, an ATM target analysed according to Jacquemin et al 2012[9]. ATM mutation detection rate in A-T patients has been estimated to be 96% (292 mutations found in a series of 305 alleles tested)[10].

**Magnetic resonance imaging**

All MRIs included T1, T2-weighted and FLAIR images. Axial, sagittal and coronal images were performed. Cerebellar atrophy was considered on MRI sagittal and axial slides by both a neuroradiologist and a neurologist.

**Nerve conduction study**

Motor and sensory nerve conduction studies (NCS) were performed with a Medtronic KEYPOINT device (Alpine Biomed, USA). Skin impedance was maintained below 5KΩ and limb temperature at 32°C. Motor nerves examination included at least bilateral peroneal and right posterior tibial nerves at the lower limbs (LL), and right median and ulnar nerves at the upper limbs (UL), using supra-maximal stimulation. Additionally, other nerves were sometimes studied. For each motor nerve, distal latency (DL), peak to baseline amplitude of the compound muscle action potentials (CMAPs), motor nerve conduction velocity (MNCV) and F-wave latency (F-WL) were recorded with classical procedure. Sensory nerves study included sural nerves at the LL bilaterally as well as right median and ulnar nerves at the UL. For each sensory nerve, distal latency (DL), peak to peak amplitude of the sensory nerve action potential (SNAP) and sensory nerve conduction velocity (SNCV) were measured. Sensory nerve action potentials (SNAPs) were recorded with an orthodromic procedure for median and ulnar nerves and antidromically for sural nerves.

**Statistical analysis**

Patients’ data were analyzed using the statistical software package Statistical Analysis System (SAS) for Windows, release 9.3 (SAS Institute Inc., Cary, NC, USA) and GraphPad Prism software, release 6.0. Categorical variables were analyzed with the χ2 test and Bonferroni-Holm correction or Fisher’s exact test when necessary. Non-parametric statistical methods were used for analysis, as most analyzed quantitative variables could not meet the assumption of normality. Such variables are presented as the median [range], while categorical variables are presented as numbers and percentage of patients (n[%]). We used unpaired two-tailed Mann Whitney t test or Kruskal–Wallis test for all comparisons of quantitative variables across groups. In case of significance, groups were pairwise compared by using Bonferroni multiple comparisons adjustment method for the p-values. Pearson or Spearman correlation coefficients were computed in order to assess the strength of the linear relationship between two quantitative variables. Median age and disease duration to wheelchair were calculated by the Kaplan–Meier method. A p value < 0.05 was considered statistically significant.

1 Schmitz-Hübsch T, du Montcel ST, Baliko L, *et al.* Scale for the assessment and rating of ataxia: development of a new clinical scale. *Neurology* 2006;**66**:1717–20. doi:10.1212/01.wnl.0000219042.60538.92

2 Anheim M, Monga B, Fleury M, *et al.* Ataxia with oculomotor apraxia type 2: clinical, biological and genotype/phenotype correlation study of a cohort of 90 patients. *Brain J Neurol* 2009;**132**:2688–98. doi:10.1093/brain/awp211

3 Méneret A, Ahmar-Beaugendre Y, Rieunier G, *et al.* The pleiotropic movement disorders phenotype of adult ataxia-telangiectasia. *Neurology* 2014;**83**:1087–95. doi:10.1212/WNL.0000000000000794

4 Tranchant C, Fleury M, Moreira MC, *et al.* Phenotypic variability of aprataxin gene mutations. *Neurology* 2003;**60**:868–70.

5 Moreira M-C, Klur S, Watanabe M, *et al.* Senataxin, the ortholog of a yeast RNA helicase, is mutant in ataxia-ocular apraxia 2. *Nat Genet* 2004;**36**:225–7. doi:10.1038/ng1303

6 Moreira MC, Barbot C, Tachi N, *et al.* The gene mutated in ataxia-ocular apraxia 1 encodes the new HIT/Zn-finger protein aprataxin. *Nat Genet* 2001;**29**:189–93. doi:10.1038/ng1001-189

7 Houdayer C, Dehainault C, Mattler C, *et al.* Evaluation of in silico splice tools for decision-making in molecular diagnosis. *Hum Mutat* 2008;**29**:975–82. doi:10.1002/humu.20765

8 Tavtigian SV, Byrnes GB, Goldgar DE, *et al.* Classification of rare missense substitutions, using risk surfaces, with genetic- and molecular-epidemiology applications. *Hum Mutat* 2008;**29**:1342–54. doi:10.1002/humu.20896

9 Jacquemin V, Rieunier G, Jacob S, *et al.* Underexpression and abnormal localization of ATM products in ataxia telangiectasia patients bearing ATM missense mutations. *Eur J Hum Genet EJHG* 2012;**20**:305–12. doi:10.1038/ejhg.2011.196

10 Micol R, Ben Slama L, Suarez F, *et al.* Morbidity and mortality from ataxia-telangiectasia are associated with ATM genotype. *J Allergy Clin Immunol* 2011;**128**:382–389.e1. doi:10.1016/j.jaci.2011.03.052

11 Le Ber I, Moreira M-C, Rivaud-Péchoux S, *et al.* Cerebellar ataxia with oculomotor apraxia type 1: clinical and genetic studies. *Brain J Neurol* 2003;**126**:2761–72. doi:10.1093/brain/awg283

12 Le Ber I, Bouslam N, Rivaud-Péchoux S, *et al.* Frequency and phenotypic spectrum of ataxia with oculomotor apraxia 2: a clinical and genetic study in 18 patients. *Brain J Neurol* 2004;**127**:759–67. doi:10.1093/brain/awh080

13 Clausi S, De Luca M, Chiricozzi FR, *et al.* Oculomotor deficits affect neuropsychological performance in oculomotor apraxia type 2. *Cortex J Devoted Study Nerv Syst Behav* 2013;**49**:691–701. doi:10.1016/j.cortex.2012.02.007

14 Panouillères M, Frismand S, Sillan O, *et al.* Saccades and eye-head coordination in ataxia with oculomotor apraxia type 2. *Cerebellum Lond Engl* 2013;**12**:557–67. doi:10.1007/s12311-013-0463-1

15 Lewis RF, Crawford TO. Slow target-directed eye movements in ataxia-telangiectasia. *Invest Ophthalmol Vis Sci* 2002;**43**:686–91.

16 Riise R, Ygge J, Lindman C, *et al.* Ocular findings in Norwegian patients with ataxia-telangiectasia: a 5 year prospective cohort study. *Acta Ophthalmol Scand* 2007;**85**:557–62. doi:10.1111/j.1600-0420.2007.00890.x

17 Shaikh AG, Marti S, Tarnutzer AA, *et al.* Gaze fixation deficits and their implication in ataxia-telangiectasia. *J Neurol Neurosurg Psychiatry* 2009;**80**:858–64. doi:10.1136/jnnp.2008.170522

18 Lewis RF, Lederman HM, Crawford TO. Ocular motor abnormalities in ataxia telangiectasia. *Ann Neurol* 1999;**46**:287–95.

**Supplementary Table 1: Detailed individual video-oculographic recording data, mutation status, SARA and SDFS scores at time of recording of AOA1, AOA2, AT and Control groups.**

**Supplementary Table 1a: Detailed individual mutation status, Age, Disease duration, SARA and SDFS scores at time of recording**

| **Patient Number**  **/Gender**  **/Origin** | **Diagnosis** | **Nucleotide change (exon)** | **Amino Acid change** | **Mutation status** | **Age at VO** | **DD at VO** | | **SDFS** | **SDFS/DD** | | **SARA** | **SARA/DD** |
| --- | --- | --- | --- | --- | --- | --- | --- | --- | --- | --- | --- | --- |
|  |  |  |  |  |  |  | |  |  | |  |  |
| 1/ F / NA | AOA1 | 837G>A (exon 6) (cf Moreira et al, Nat Genet, 2001) | W279* | Homozygous | 22.1 | 22.1 | | 5 | 0.23 | | 18 | 0.82 |
| 2/ F / Italy | AOA1 | 837G>A (exon 6) | W279* | Homozygous | 42.8 | 42.8 | | 6 | 0.14 | | 23.5 | 0.55 |
| 3/ M / North Africa | AOA1 | 875-1G>A (splice mutation on the acceptor splice site of exon 7 (cf Amouri et al 2004 Neurology)) & 809C>T (exon 6) (cf Laurencin et al 2016) | skipping of exon 7 and S270F | Compound heterozygous | 58.7 | 58.7 | | 6 | 0.10 | | 19 | 0.32 |
| 4/ M / NA | AOA1 | 336_337 delCA (exon3) & 617C>T (exon5) | frame shift H112Qfs*10 & P206L | Compound heterozygous | 40 | 40 | | 3 | 0.08 | | 12 | 0.30 |
| 5/ F / NA | AOA1 | 837G>A (exon 6) | W279* | Homozygous | 18.1 | 18.1 | | 5 | 0.28 | | 23 | 1.27 |
| 6/ M / North Africa | AOA1 | 875-1G>A (splice mutation on the acceptor splice site of exon 7 (cf Amouri et al 2004 Neurology)) | skipping of exon 7 | Homozygous | 31.2 | 31.2 | | 6 | 0.19 | | 28 | 0.90 |
| 7/ M / North Africa | AOA1 | 875-1G>A (splice mutation on the acceptor splice site of exon 7 (cf Amouri et al 2004 Neurology)) | skipping of exon 7 | Homozygous | 15.4 | 15.4 | | ND | ND | | ND | ND |
| 8/ F / France | AOA1 | 837G>A (exon 6) (cf Moreira et al, Nat Genet, 2001) | W279* | Homozygous | 31.6 | 31.6 | | 5 | 0.16 | | 26.5 | 0.84 |
| 9/ F / France | AOA1 | 837G>A (exon 6) (cf Moreira et al, Nat Genet, 2001) | W279* | Homozygous | 44 | 44 | | 6 | 0.14 | | 35 | 0.79 |
| 10/ M / France | AOA1 | 837G>A (exon 6) (cf Moreira et al, Nat Genet, 2001) | W279* | Homozygous | 46.9 | 47 | | 6 | 0.13 | | 34 | 0.72 |
| 11/ M / France | AOA1 | 837G>A (exon 6) & 589A>C (exon5)  (cf Tranchant et al. 2003) | W279* & K197Q | Compound heterozygous | 35.7 | 35.7 | | 5 | 0.14 | | 23.5 | 0.66 |
| 12/ F / France | AOA1 | 837G>A (exon 6) & 589A>C (exon5)  (cf Tranchant et al. 2003) | W279* & K197Q | Compound heterozygous | 38.2 | 38.2 | | 5 | 0.13 | | 22.5 | 0.59 |
| 13/ M / France | AOA2 | 6017G>A (exon 13)  7319A>G (exon 24) | C2006Y & D2440G | Compound heterozygous | 26.6 | 26.6 | | 5 | 0.19 | | 30 | 1.13 |
| 14/ M / France | AOA2 | 5929C>T (exon 12) & 7000_7012del13insT (exon 21)  (cf Anheim et al 2009) | L1977F & R2334_S2338delinsC | Compound heterozygous | 37.6 | 37.6 | | 6 | 0.16 | | 20 | 0.53 |
| 15/ M / France | AOA2 | 5929C>T (exon 12) & 7000_7012del13insT (exon 21)  (cf Anheim et al 2009) | L1977F & R2334_S2338delinsC | Compound heterozygous | 37.6 | 37.6 | | 6 | 0.16 | | 20.5 | 0.55 |
| 16/ F / France | AOA2 | 994C>T (exon 6) & 2966_2970delGGAAA (exon 8)  (cf Moreira et al 2004) | R332W & frame shift R989Sfs*5 | Compound heterozygous | 47.3 | 47.3 | | 6 | 0.13 | | 19 | 0.40 |
| 17/ M / France | AOA2 | 4075C>T (exon 8) & 6694C>G (exon 19) | Q1359* & R2232C | Compound heterozygous | 24.7 | 24.7 | | 3 | 0.12 | | 18 | 0.73 |
| 18/ M / France | AOA2 | 6547G>A (exon 18)  (cf Anheim et al 2009) | A2183T | Homozygous | 51 | 51 | | 5 | 0.10 | | 23 | 0.45 |
| 19/ M / France | AOA2 | 6547G>A (exon 18)  (cf Anheim et al 2009) | A2183T | Homozygous | 50.6 | 50.6 | | 6 | 0.12 | | 23 | 0.45 |
| 20/ M / North Africa | AOA2 | 7010T>A (exon 21) | V2337D | Homozygous | 41.7 | 41.7 | | 3 | 0.07 | | 17.5 | 0.42 |
| 21/ M / North Africa | AOA2 | 4144C>T (exon 8) | Q1382* | Homozygous | 21.5 | 21.5 | | 6 | 0.28 | | 25.5 | 1.18 |
| 22/ M / North Africa | AOA2 | 4144C>T (exon 8) | Q1382* | Homozygous | 21.5 | 21.5 | | 6 | 0.28 | | 24.5 | 1.14 |
| 23/ F / France | AOA2 | 994C>T (exon 6) & 2966_2970delGGAAA (exon 8) | R332W & frame shift R989Sfs*5 | Compound heterozygous | 44.8 | 44.8 | | 6 | 0.13 | | 22 | 0.49 |
| 24/ M / North Africa | AT | 9022C>T | R3008C | Homozygous | 25.3 | 25.3 | | 6 | 0.24 | | 28 | 1.11 |
| 25/ M / North Africa | AT | 9022C>T | R3008C | Homozygous | 24.5 | 24.5 | | 6 | 0.24 | | 27 | 1.10 |
| 26/ F / France | AT | IVS10-6T>G (intron 10) & 8624A>G (exon 61) | Possible abnormal splicing & N2875S | Compound heterozygous | 52.4 | 52.4 | | 4 | 0.08 | | 18 | 0.34 |
| 27/ M / France | AT | IVS28-1G>C & IVS34+32insAlu | skipping of exon 28 & stop codon insertion | Compound heterozygous | 36.5 | 36.5 | | 6 | 0.16 | | 22.5 | 0.62 |
| 28/ F / France | AT | IVS19+2T>G & 8147T>C | abnormal splicing & V2716A | Compound heterozygous | 28.6 | 28.6 | | 2 | 0.07 | | 12 | 0.42 |
| 29/ M / France | AT | 824delT & 3248A>G | L275* & H1083R | Compound heterozygous | 23.1 | 23.1 | | 2 | 0.09 | | 13.5 | 0.58 |
| 30/ F / France | AT | IVS10-6T>G (intron 10) & 8624A>G (exon 61) | Possible abnormal splicing & N2875S | Compound heterozygous | 44.3 | 44.3 | | 3 | 0.07 | | 18 | 0.41 |
| 31/ F / Caucasian | AT | 7456C>T & 8161G>A | R2486* & D2721N | Compound heterozygous | 24.2 | 24.2 | | 6 | 0.25 | | 26 | 1.07 |
| 32/ M / Caucasian | AT | 7456C>T & 8161G>A | R2486* & D2721N | Compound heterozygous | 23 | 23 | | 6 | 0.26 | | 25 | 1.09 |
| 33/ F / France | AT | IVS19+2T>G & 8147T>C | abnormal splicing & V2716A | Compound heterozygous | 21.9 | 21.9 | | 4 | 0.18 | | 20.5 | 0.93 |
| 34/ F / France | AT | IVS19+2T>G & 8147T>C | abnormal splicing & V2716A | Compound heterozygous | 28.1 | 28.1 | | 4 | 0.14 | | 20 | 0.71 |
| 35/ F / France | AT | IVS19+2T>G & 8147T>C | abnormal splicing & V2716A | Compound heterozygous | 31 | 31 | | 1 | 0.03 | | 7.5 | 0.24 |
| 36/ F / France &North Africa | AT | IVS21+1G>A (exon 21) & IVS55+5delG (exon 55) | 2838del83/955* & 1788del139/2599* | Compound heterozygous | 35.4 | 35.4 | | 6 | 0.17 | | 26.5 | 0.75 |
| 37/ M / NA | AT | dupEx64+65 & 6108T>G | frame-shift & Y2036* | Compound heterozygous | 27.1 | 27.1 | | 6 | 0.22 | |  |  |
| 38/ M / France | AT | 6680A>G & 2 UV IVS36-10T>G & 7475T>G | R2227K & L2492R. Functional testing : no ATM protein, no KAp1 phosphorylation (ATM specific) | Compound heterozygous | 49.4 | 49.4 | | 1 | 0.02 | | 9 | 0.18 |
| 39/ F / France | AT | 6680A>G & 2 UV IVS36-10T>G & 7475T>G | R2227K & L2492R. Functional testing : no ATM protein, no KAp1 phosphorylation (ATM specific) | Compound heterozygous | 49.3 | 49.3 | | 4 | 0.08 | | 20 | 0.41 |
| 40/ F Czech &Poland | AT | 3848T>C & 6661G>A | L1283P & E2221K | Compound heterozygous | 54.8 | 54.8 | | 7 | 0.13 | | 33 | 0.60 |
| C1/ F |  |  |  |  | 30 |  | |  |  | |  |  |
| C2/ F |  |  |  |  | 33 |  | |  |  | |  |  |
| C3/ F |  |  |  |  | 38 |  | |  |  | |  |  |
| C4/ F |  |  |  |  | 34 |  | |  |  | |  |  |
| C5/ F |  |  |  |  | 36 |  | |  |  | |  |  |
| C6/ M |  |  |  |  | 32 |  | |  |  | |  |  |
| C7/ F |  |  |  |  | 36 |  | |  |  | |  |  |
| C8/ M |  |  |  |  | 33 |  | |  |  | |  |  |
| C9/ F |  |  |  |  | 34 |  | |  |  | |  |  |
| C10/ M |  |  |  |  | 36 |  |  | |  |  | |  |
| C11/ M |  |  |  |  | 35 |  |  | |  |  | |  |
| C12/ F |  |  |  |  | 33 |  |  | |  |  | |  |
| C13/ M |  |  |  |  | 33 |  |  | |  |  | |  |
| C14/ M |  |  |  |  | 33 |  |  | |  |  | |  |
| C15/ M |  |  |  |  | 36 |  |  | |  |  | |  |
| C16/ F |  |  |  |  | 30 |  |  | |  |  | |  |
| C17/ F |  |  |  |  | 25 |  |  | |  |  | |  |

**Supplementary Table 1b: Detailed individual video-oculographic recording data**

| Patient Number | FIXATION | | | SACCADES | | | | | | | | Antisaccades | | Smooth pursuit |
| --- | --- | --- | --- | --- | --- | --- | --- | --- | --- | --- | --- | --- | --- | --- |
|  |  |  |  | Latency (mean ± SD) | | | | Gain (horizontal) | | Velocity | | Error Rate (%) | |  |
|  | SWJ | DBN | GEN | Left | Right | Upward | Downward | Centrifugal | Centripetal | Horiz | Vertic | Target R | Target L |  |
| **AOA1** |  |  |  |  |  |  |  |  |  |  |  |  |  |  |
| 1 | 0 | 1 | 1 | 442±189 | 435±84 | 326±135 | 237±60 | Hyper | Hyper | 0 | 0 | 50 | 58 | 1 |
| 2 | 0 | 0 | 0 | 200±49 | 146±90 | ND | ND | Hypo | Hypo | 1 | 1 | 0 | 0 | 1 |
| 3 | 0 | 0 | 0 | 191±64 | 186±29 | 293±122 | 181±48 | Hypo | N | 0 | 0 | 25 | 25 | ND |
| 4 | 1 | 0 | 1 | 145±27 | 134±28 | 154±27 | 156±26 | Hypo | Hyper | 0 | 0 | 19 | 25 | 0 |
| 5 | 1 | 0 | 1 | 245±55 | 204±41 | 245±194 | 263±142 | Hypo | Hypo | 0 | 0 | 75 | 80 | 1 |
| 6 | 0 | 0 | 1 | NA | NA | NA | NA | Hypo | Hypo | 1 | 0 | ND | ND | 1 |
| 7 | 0 | 0 | 0 | NA | NA | NA | NA | ND | ND | 1 | 1 | ND | ND | 1 |
| 8 | 0 | 1 | 1 | 250±134 | 492±73 | 241±68 | 221±44 | Hypo | Hypo | 0 | 0 | 60 | 7 | 1 |
| 9 | 1 | 1 | 1 | 171±47 | 151±44 | 179±27 | 193±55 | Hypo | Hyper | 0 | 0 | ND | ND | 1 |
| 10 | 1 | 0 | 1 | 165±55 | 179±50 | 180±52 | 192±60 | Hypo | N | 0 | 0 | ND | ND | 1 |
| 11 | 0 | 0 | 0 | 216±72 | ND | ND | ND | Hypo | Hypo | 1 | 1 | 0 | 0 | 1 |
| 12 | 0 | 0 | 0 | 212±20 | 246±64 | ND | ND | Hyper | Hyper | 0 | 0 | 0 | 0 | 1 |
| **AOA2** |  |  |  |  |  |  |  |  |  |  |  |  |  |  |
| 13 | 1 | 0 | 1 | 185±62 | 199±49 | 221±82 | 253±54 | Hypo | Hyper | 0 | 1 | 38 | 56 | 1 |
| 14 | 1 | 1 | 1 | 132±16 | 165±51 | 202±76 | 141±27 | Hypo | Hyper | 0 | 1 | 56 | 47 | 1 |
| 15 | 1 | 1 | 1 | 172±44 | 223±70 | 287±113 | 171±30 | Hypo | Hyper | 0 | 1 | 6 | 6 | 1 |
| 16 | 1 | 1 | 1 | 194±29 | 189±36 | 335±84 | 218±27 | N | Hyper | 0 | 0 | 69 | 69 | 1 |
| 17 | 1 | 0 | 0 | 151±40 | 121±17 | 137±32 | 150±35 | Hypo | Hyper | 0 | 0 | 56 | 50 | 1 |
| 18 | 1 | 1 | 1 | 255±104 | 241±75 | 201±20 | 287±110 | N | Hyper | 0 | 0 | 75 | 56 | 1 |
| 19 | 0 | 0 | 1 | 190±32 | 236±52 | 283±101 | 261±135 | Hypo | Hyper | 0 | 0 | 63 | 44 | 1 |
| 20 | 1 | 1 | 0 | 354±22 | 268±106 | 322±142 | 270±124 | Hyper | Hyper | 0 | 0 | 21 | 10 | 1 |
| 21 | 0 | 1 | 1 | 174±39 | 169±62 | 133 ±16 | 131±16 | Hypo | Hypo | 1 | 1 | 71 | 19 | 1 |
| 22 | 0 | 1 | 1 | 131±20 | 148±37 | 160±22 | 152±12 | Hypo | Hypo | 1 | 1 | 80 | 81 | 1 |
| 23 | 0 | 0 | 1 | 150±38 | 157±39 | 295±101 | 185±33 | Hypo | Hypo | 0 | 1 | 53 | 47 | 1 |
| **AT** |  |  |  |  |  |  |  |  |  |  |  |  |  |  |
| 24 | 0 | ND | 1 | 449±159 | 362±142 | ND | ND | Hypo | Hypo | 0 | ND | ND | ND | 1 |
| 25 | 0 | 0 | 1 | 251±89 | 145±21 | ND | ND | Hypo | Hypo | 0 | 1 | 72 | 58 | 1 |
| 26 | 1 | 1 | 1 | 163±16 | 172±37 | 292±39 | 196±31 | Hypo | Hypo | 1 | 1 | 73 | 100 | 1 |
| 27 | 0 | 1 | 1 | 300±101 | 346±173 | ND | ND | Hypo | N | 0 | 0 | 27 | 44 | 1 |
| 28 | 0 | ND | 0 | 141±64 | 180±60 | ND | ND | Hypo | Hyper | 0 | ND | 50 | 41 | 0 |
| 29 | 0 | 0 | 0 | 193±37 | 149±35 | 215±87 | 161±68 | Hypo | Hypo | 0 | 0 | 47 | 19 | 0 |
| 30 | 1 | 1 | 1 | 171±31 | 180±66 | 249±115 | 196±39 | Hypo | Hyper | 0 | 0 | 25 | 37 | 1 |
| 31 | 0 | 1 | 1 | 213±76 | 219±71 | 252±57 | 198±54 | Hypo | Hypo | 0 | 0 | 60 | 80 | 1 |
| 32 | 0 | 0 | 1 | 220±117 | 288±53 | 153±17 | 177±79 | Hypo | Hypo | 0 | 0 | 94 | 75 | 1 |
| 33 | 1 | 0 | 0 | 274±86 | 208±67 | 302±58 | 266±47 | Hypo | Hyper | 0 | 0 | 16 | 25 | 0 |
| 34 | 0 | 0 | 0 | 170±53 | 164±29 | 239±154 | 241±74 | Hyper | Hyper | 0 | 0 | 25 | 19 | 1 |
| 35 | 1 | 0 | 0 | 206±31 | 224±20 | 168±36 | 184±42 | Hyper | Hyper | 0 | 0 | 6 | 6 | 0 |
| 36 | 0 | 1 | 1 | 167±37 | 168±38 | 244±40 | 204±39 | Hypo | Hyper | 0 | 0 | ND | ND | 1 |
| 37 | ND | ND | ND | ND | ND | ND | ND | Hypo | Hypo | ND | 1 | ND | ND | 1 |
| 38 | 1 | 0 | 0 | 170±17 | 183±16 | 221±34 | 184±35 | Hypo | N | 0 | 0 | 50 | 80 | 1 |
| 39 | 0 | 0 | 0 | 179±37 | 220±36 | 197±39 | 309±64 | N | N | 0 | 0 | 87 | 81 | 1 |
| 40 | 0 | 1 | 0 | ND | ND | ND | ND | Hypo | Hypo | 0 | 0 | ND | ND | ND |
| **Controls** |  |  |  |  |  |  |  |  |  |  |  |  |  |  |
| C1 | 0 | 0 | 0 | 157±30 | 149±25 | 152±39 | 165±22 | N | N | 0 | 0 | 0 | 0 | 0 |
| C2 | 0 | 0 | 0 | 236±70 | 189±68 | 221±50 | 249±46 | N | N | 0 | 0 | ND | ND | 0 |
| C3 | 0 | 0 | 0 | 156±24 | 150±34 | 193±81 | 184±50 | N | N | 0 | 0 | ND | ND | 0 |
| C4 | 0 | 0 | 0 | 245±109 | 224±97 | 177±64 | 232±125 | N | N | 0 | 0 | 13 | 6 | 0 |
| C5 | 0 | 0 | 0 | 183±27 | 170±15 | 174±27 | 176±38 | N | N | 0 | 0 | 13 | 8 | 0 |
| C6 | 0 | 0 | 0 | 155±31 | 170±37 | 182±49 | 177±29 | N | N | 0 | 0 | 19 | 6 | 0 |
| C7 | 0 | 0 | 0 | 145±36 | 154±97 | 168±51 | 210±69 | N | N | 0 | 0 | 44 | 20 | 0 |
| C8 | 0 | 0 | 0 | 176±27 | 165±38 | 185±35 | 161±39 | N | N | 0 | 0 | 0 | 0 | 0 |
| C9 | 0 | 0 | 0 | 165±38 | 244±57 | 153±51 | 162±34 | N | N | 0 | 0 | 0 | 6 | 0 |
| C10 | 0 | 0 | 0 | 139±25 | 152±26 | ND | 187±15 | N | N | 0 | 0 | 25 | 20 | 0 |
| C11 | 0 | 0 | 0 | 189±26 | 200±29 | 193±41 | 190±25 | N | N | 0 | 0 | 0 | 19 | 0 |
| C12 | 0 | 0 | 0 | 136±19 | 141±10 | ND | ND | N | N | 0 | 0 | 40 | 7 | 0 |
| C13 | 0 | 0 | 0 | 157±16 | 175±45 | 156±28 | 169±25 | N | N | 0 | 0 | 14 | 12 | 0 |
| C14 | 0 | 0 | 0 | ND | ND | 162±28 | 147±51 | N | N | 0 | 0 | 20 | 25 | 0 |
| C15 | 0 | 0 | 0 | 193±36 | 193±35 | 171±23 | 188±34 | N | N | 0 | 0 | 6 | 0 | 0 |
| C16 | 0 | 0 | 0 | 170±31 | 195±36 | 176±23 | 160±33 | N | N | 0 | 0 | 12 | 25 | 0 |
| C17 | 0 | 0 | 0 | 190±43 | 175±38 | 210±53 | 202±49 | N | N | 0 | 0 | 0 | 6 | 0 |

Latencies are expressed in ms.

Age and DD are expressed in years.

0 means parameter (Smooth pursuit, Velocity) is normal or sign (SWJ, DBN, GEN) is absent, 1 means parameter (Smooth pursuit, Velocity) is altered or sign (SWJ, DBN, GEN) is present.

C1 to C17 are healthy subjects, controls 1 to 17.

Cases 26 and 30 are two sisters presenting with the same neurological disorder. They are both carrying the same ATM variants, each exclusively located in one allele. The c.8224A>G ;p.(N2875S) variant may be causal according to a A-GVGD score of 45. The IVS10-6T>G variant may be associated with a splice defect. The ATM expression in a lymphoblastoid cell line derived from individual 30 is half the expected level. It has been concluded that ATM is the likely locus of the disease.

Abbreviations: AOA1: ataxia with oculomotor apraxia type 1; AOA2: ataxia with oculomotor apraxia type 2; AT: Ataxia Telangiectasia; DBN: down beat nystagmus; DD: Disease Duration; F: female; GEN : gaze-evoked nystagmus; Horiz: horizontal; Hyper: hypermetric; Hypo: hypometric; L: left; M: male; N: normal; NA: data not available; ND: not done; R: right; SARA: Scale for the Assessment and Rating of Ataxia; SDFS: Spinocerebellar degeneration functional score; SWJ: Square Waves Jerks; Vertic: vertical; VO: video-oculography.

**Supplementary Table 2: AOA1, AOA2 and AT patient demographics**

|  | **AOA1** | **AOA2** | **AT** |  |
| --- | --- | --- | --- | --- |
| **n=** | **12** | **11** | **17** | ***p*** |
| **Sex ratio M:F** | 1 | 4.5 | 0.7 | 0.1365 |
| **Origin** |  |  |  |  |
| France | 5 (55.6) | 8 (72.7) | 11 (68.8) |  |
| North Africa | 3 (33.3) | 3 (27.3) | 3 (18.8) |  |
| Eastern Europe |  |  | 1 (6.3) |  |
| Italy | 1 (11.1) |  |  |  |
| Caucasian unspecified |  |  | 2 (12.5) |  |
| ND | 3 (25) |  | 1 (5.9) |  |
| **Sign at onset** |  |  |  |  |
| Unsteadiness | 12 (100) | 5 (62.5) | 10 (83.3) |  |
| Clumsiness | 1 (8.3) | 3 (37.5) |  |  |
| Dysarthria | 1 (8.3) | 1 (12.5) | 2 (16.7) |  |
| Swallowing disturbances |  | 1 (12.5) |  |  |
| Diplopia |  | 1 (12.5) | 1 (8.3) |  |
| Dystonia |  |  | 2 (16.7) |  |
| ND | 0 | 3 (27.3) | 5 (29.4) |  |

Categorical variables are expressed as numbers and percentages [n(%)] and continuous variables as median [range].

Total percentages of initial symptoms can exceed 100% because multiple symptoms could appear simultaneously.

Abbreviations: AOA1: ataxia with oculomotor apraxia type 1, AOA2: ataxia with oculomotor apraxia type 2, AT: Ataxia-Telangiectasia; F: Female; M: Male; ND: Not determined.

**Supplementary Table 3: Investigations: biomarkers, nerve conduction and imaging findings**

|  | **AOA1** | **AOA2** | **AT** | **Controls** |  |
| --- | --- | --- | --- | --- | --- |
| **n=** | **12** | **11** | **17** | **100** | ***p*** |
| **MRI** |  |  |  |  |  |
| Cerebellar atrophy | 7/7 (100) | 11/11 (100) | 15/15 (100) |  |  |
| **NCS** |  |  |  |  |  |
| Sensory motor neuropathy | 7/8 (87.5) | 9/10 (90) | 13/17 (76.5) |  | 0.6161 |
| **Laboratory findings** | | | | |  |
| **Albumin** serum level | 33.35 [25-41]* ͣ | 40.2 [37.5-47.7]* | 44 [31-47] ͣ |  | *<0.05  ͣ<0.0001 |
| Decreased albumin level | 6 (50) | 0 | 1 (7) |  | <0.01 |
| **AFP** serum level (µg/L) | 7.7 [2.1-13.6]*ᵇᵈ | 38.9 [22-300.9]*^c^ | 88 [4.5-464.9]^c^ᵈ | 3.4 [0.8-17] | *andᵇ<0.05  ^c^<0.0001  ᵈ<0.01 |
| Increased AFP | 7 (63.6) ^e^ | 9 (90) ^c^ | 16 (94.1) ^c^ | 10 (10) | ^c^<0.0001  ^e^<0.001 |
| Abnormal **Karyotype** |  |  | 4 (25) |  |  |

Brain MRI was performed in 83% (n=33/40) of patients with respectively 7/12 AOA1, 11/11 AOA2 and 15/17 AT patients investigated.

Motor and sensory nerve conduction studies (NCS) were performed in 90 % (n=36/40) of patients with respectively 8/12 AOA1, 10/11 AOA2 and 17/17 AT patients investigated..

Categorical variables are expressed as the ratio of the number of patients presenting the symptom to the total number of patients assessed and as percentages [n/N (%)]; and continuous variables as median [range].

* Significant differences after Bonferroni-Holm post-test between the AOA1 and AOA2 groups.

ͣ and ᵈ Significant differences after Bonferroni-Holm post-test between the AOA1 and AT groups.

ᵇ^, c^ and ^e^ Significant differences after Bonferroni-Holm post-test relative to the control group.

Abbreviations: AFP: Alpha-foeto protein; AOA1: ataxia with oculomotor apraxia type 1, AOA2: ataxia with oculomotor apraxia type 2, AT: Ataxia-Telangiectasia; NCS: Nerve conduction studies

**Supplementary Table 4: Literature review of video-oculographic findings in AOA1, AOA2, or AT patients**

| **Number & type of oculographic recordings**  **/ Type of ataxia** | **Age (y)** | **Fixation** | | | | **Horizontal Saccades** | | | | **Vertical Saccades** | **Saccadic Pursuit** | **AS** | **Reference** |
| --- | --- | --- | --- | --- | --- | --- | --- | --- | --- | --- | --- | --- | --- |
|  |  | **SWJ** | **DBN** | **GEN** | **abNl** | **↑**  **latency** | **Gain / Amplitude** | | **Velocity** |  |  |  |  |
|  |  |  |  |  |  |  | Hypo | Hyper |  |  |  |  |  |
| **3 VO**  **/ AOA1** | 25.3 (22-29) |  |  |  |  | Not specified except: 1 OMA, 2 no OMA | | | |  |  |  | Tranchant et al [4] 2003 |
| **6 EOG**  **/ AOA1** | NA | 6 (100) |  | 6  (100) | 6  (100) | 3  (50) | +++ |  | normal if small saccades of 5° amplitude |  |  | ↑ errors  (38% vs 4.3%  in ctls) | Le Ber et al [11] 2003 |
| **7 EOG**  **/ AOA2** | NA |  |  | 89%  Clinical  exam |  | 3  (43) | +++ |  | overall mean normal (360°/s  vs  335°/s) |  | 100% Clinical  exam | ↑ errors  (40% vs 4%  in ctls) | Le Ber et al [12] 2004 |
| **2 VO**  **/ AOA2** | 39  (38-40) | 2 (100) |  | 1  (50) | 2  (100) | 2  (100)  at 238ms & 249ms |  |  |  |  |  |  | Clausi et al [13] 2013 |
| **5 VO**  **/ AOA2** | 31  (23-41) |  |  |  |  | Yes in memory-guided saccades | 4/5  (80) |  | slower reactive & scanning saccades in patients vs ctls |  |  | ↑ errors  (36% vs 3%  in ctls) | Panouillères et al [14] 2013 |
| **3 coils**  **/ AT** | NA  (15-23) |  |  |  |  |  | +++ |  | appropriate for the amplitude, & slow movement before or after initial saccade |  |  |  | Lewis et al [15] 2002 |
| **10 VO**  **/ AT** | 9.8  (2-22) | ++ |  | ++ | 10  (100) | 10  (100) | +++ |  |  |  | 10  (100) |  | Riise et al [16] 2007 |
| **13 (7 VO, 5 coils)**  **/ AT** | 26  (13-54) | 11/13 (85) | ++ (n=6  & 1 UBN) | ++ | 13/13 (100) |  |  |  |  |  |  |  | Shaikh et al [17] 2009 |
| **14 VO**  **/ AT** | 27.4  (20-36) |  | 2/14  (14) | 6/14  (43) |  | 5/12  (42) | 11/13  (85) | 5/13  (38) | ↓ in 3/12  (25) |  | 13  (100) | 9/10  (90) | Méneret et al [3] 2014 |
| **33 EOG / AT** | 10.2  (3-25) | (36%) | (4%) | (4%) | +++ | (77%) | ++ |  | normal or too high for amplitude |  | +++ |  | Lewis et al[18] 1999 |
| **57 VO = 40 AOA1 AOA2 & AT + 17 ctls** | 35.2  (15-59) |  |  |  |  |  |  |  |  |  |  |  | Current study |
| **AOA1** |  | 4 (33.3) | 3  (25) | 7  (58.3) | 8  (58.3) | 4  (40) | 9  (81.8) | 2  (18.2) | ↓ in 4  (33.3) |  | 10  (91) | 23.5  [0-77.5] | Current study |
| **AOA2** |  | 7 (63.6) | 7  (63.6) | 9  (81.8) | 11  (100) | 4  (36.4) | 8  (72.7) | 1  (9.1) | ↓ in 2 (18.2) | ↓ velocity | 11  (100) | 51.5  [6-80.5] | Current study |
| **AT** |  | 5 (31.3) | 6  (42.9) | 8  (50) | 13  (75) | 7  (46.7) | 14  (82.4) | 2  (11.8) | ↓ in 1 (6.3) | ↑ upward latency | 12  (75) | 45.5  [6-86.5] | Current study |

Categorical variables are expressed as numbers and percentages (n(%)) and continuous variables as median [range] when data is available. When semi-quantitative data is available then + is present or mild, ++ is frequent or moderate; +++ is very frequent or intense.

Abbreviations: ↑: increased; ↓: decreased; abNl : abnormal; AOA1: ataxia with oculomotor apraxia type 1, AOA2: ataxia with oculomotor apraxia type 2, AS: Antisaccades; AT: Ataxia Telangiectasia; ctls : controls; DBN : down beat nystagmus; GEN : gaze-evoked nystagmus; Hyper: Hypermetric horizontal saccades; Hypo: Hypometric horizontal saccades; NA: data not available; ND: Not done; OMA: Oculo-motor apraxia; SWJ: Square Waves Jerks, UBN : up beat nystagmus ;VO: video-oculography; vs: versus

1 Schmitz-Hübsch T, du Montcel ST, Baliko L, *et al.* Scale for the assessment and rating of ataxia: development of a new clinical scale. *Neurology* 2006;**66**:1717–20. doi:10.1212/01.wnl.0000219042.60538.92

2 Anheim M, Monga B, Fleury M, *et al.* Ataxia with oculomotor apraxia type 2: clinical, biological and genotype/phenotype correlation study of a cohort of 90 patients. *Brain J Neurol* 2009;**132**:2688–98. doi:10.1093/brain/awp211

3 Méneret A, Ahmar-Beaugendre Y, Rieunier G, *et al.* The pleiotropic movement disorders phenotype of adult ataxia-telangiectasia. *Neurology* 2014;**83**:1087–95. doi:10.1212/WNL.0000000000000794

4 Tranchant C, Fleury M, Moreira MC, *et al.* Phenotypic variability of aprataxin gene mutations. *Neurology* 2003;**60**:868–70.

5 Moreira M-C, Klur S, Watanabe M, *et al.* Senataxin, the ortholog of a yeast RNA helicase, is mutant in ataxia-ocular apraxia 2. *Nat Genet* 2004;**36**:225–7. doi:10.1038/ng1303

6 Moreira MC, Barbot C, Tachi N, *et al.* The gene mutated in ataxia-ocular apraxia 1 encodes the new HIT/Zn-finger protein aprataxin. *Nat Genet* 2001;**29**:189–93. doi:10.1038/ng1001-189

7 Houdayer C, Dehainault C, Mattler C, *et al.* Evaluation of in silico splice tools for decision-making in molecular diagnosis. *Hum Mutat* 2008;**29**:975–82. doi:10.1002/humu.20765

8 Tavtigian SV, Byrnes GB, Goldgar DE, *et al.* Classification of rare missense substitutions, using risk surfaces, with genetic- and molecular-epidemiology applications. *Hum Mutat* 2008;**29**:1342–54. doi:10.1002/humu.20896

9 Jacquemin V, Rieunier G, Jacob S, *et al.* Underexpression and abnormal localization of ATM products in ataxia telangiectasia patients bearing ATM missense mutations. *Eur J Hum Genet EJHG* 2012;**20**:305–12. doi:10.1038/ejhg.2011.196

10 Micol R, Ben Slama L, Suarez F, *et al.* Morbidity and mortality from ataxia-telangiectasia are associated with ATM genotype. *J Allergy Clin Immunol* 2011;**128**:382–389.e1. doi:10.1016/j.jaci.2011.03.052

11 Le Ber I, Moreira M-C, Rivaud-Péchoux S, *et al.* Cerebellar ataxia with oculomotor apraxia type 1: clinical and genetic studies. *Brain J Neurol* 2003;**126**:2761–72. doi:10.1093/brain/awg283

12 Le Ber I, Bouslam N, Rivaud-Péchoux S, *et al.* Frequency and phenotypic spectrum of ataxia with oculomotor apraxia 2: a clinical and genetic study in 18 patients. *Brain J Neurol* 2004;**127**:759–67. doi:10.1093/brain/awh080

13 Clausi S, De Luca M, Chiricozzi FR, *et al.* Oculomotor deficits affect neuropsychological performance in oculomotor apraxia type 2. *Cortex J Devoted Study Nerv Syst Behav* 2013;**49**:691–701. doi:10.1016/j.cortex.2012.02.007

14 Panouillères M, Frismand S, Sillan O, *et al.* Saccades and eye-head coordination in ataxia with oculomotor apraxia type 2. *Cerebellum Lond Engl* 2013;**12**:557–67. doi:10.1007/s12311-013-0463-1

15 Lewis RF, Crawford TO. Slow target-directed eye movements in ataxia-telangiectasia. *Invest Ophthalmol Vis Sci* 2002;**43**:686–91.

16 Riise R, Ygge J, Lindman C, *et al.* Ocular findings in Norwegian patients with ataxia-telangiectasia: a 5 year prospective cohort study. *Acta Ophthalmol Scand* 2007;**85**:557–62. doi:10.1111/j.1600-0420.2007.00890.x

17 Shaikh AG, Marti S, Tarnutzer AA, *et al.* Gaze fixation deficits and their implication in ataxia-telangiectasia. *J Neurol Neurosurg Psychiatry* 2009;**80**:858–64. doi:10.1136/jnnp.2008.170522

18 Lewis RF, Lederman HM, Crawford TO. Ocular motor abnormalities in ataxia telangiectasia. *Ann Neurol* 1999;**46**:287–95.

**Supplementary Figure:**  Severity of disease progression in AOA1, AOA2 and AT patients


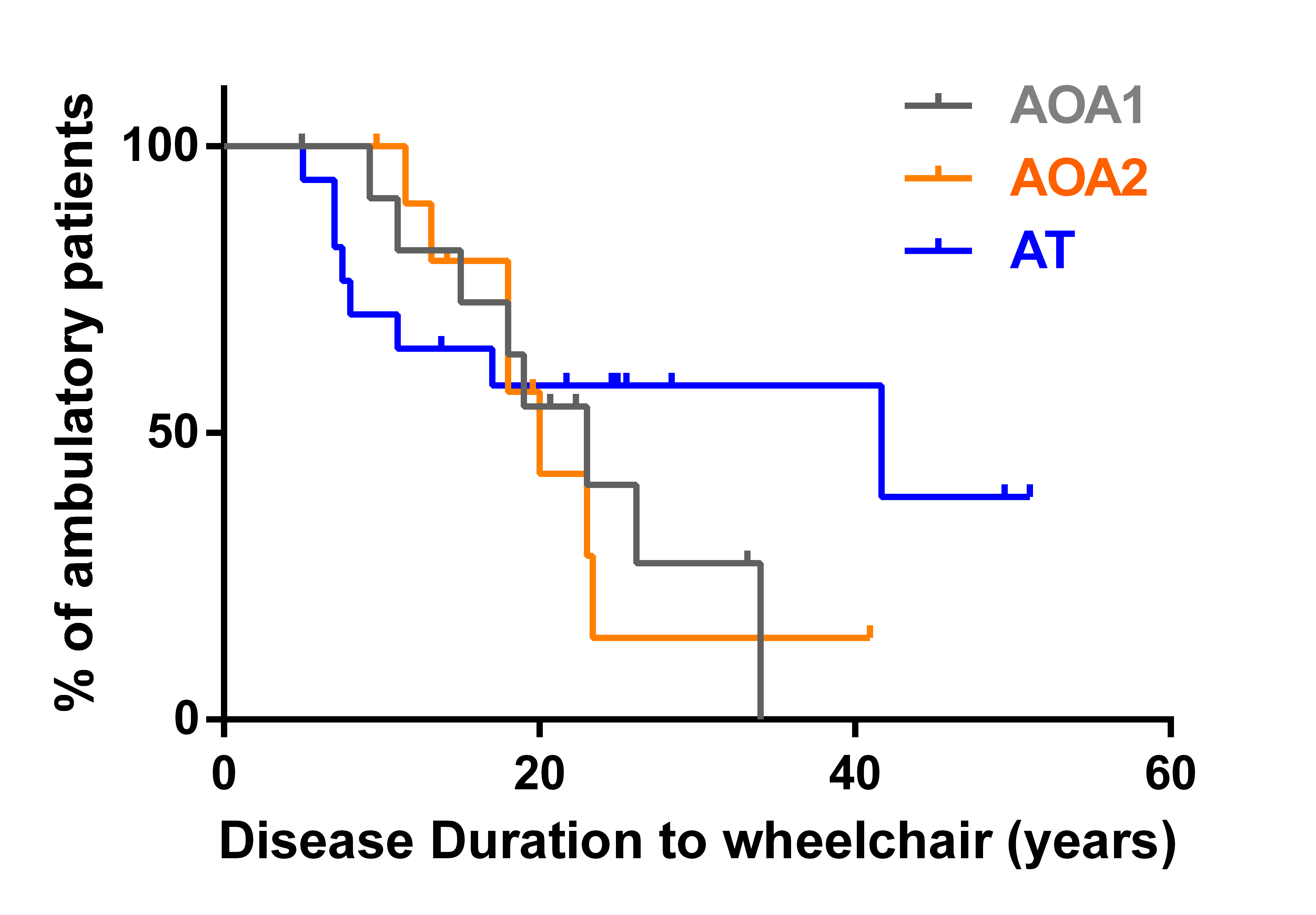


Survival curves (Kaplan–Meier) of disease duration to wheelchair in AOA1, AOA2 and AT patients are expressed in years following disease onset. All patients experienced cerebellar ataxia with an important functional disability reflecting the severity of the disease.

Abbreviations: AOA1: ataxia with oculomotor apraxia type 1; AOA2: ataxia with oculomotor apraxia type 2; AT: Ataxia Telangiectasia
